# Reduced neurobehavioral functioning in agricultural workers and rural inhabitants exposed to pesticides in northern Chile and its association with blood biomarkers inhibition

**DOI:** 10.1186/s12940-020-00634-6

**Published:** 2020-07-22

**Authors:** Muriel Ramírez-Santana, Liliana Zúñiga-Venegas, Sebastián Corral, Nel Roeleveld, Hans Groenewoud, Koos Van der Velden, Paul T. J. Scheepers, Floria Pancetti

**Affiliations:** 1 Departemento de Salud Pública, Facultad de Medicina, Universidad Católica del Norte, Coquimbo, Chile; 2 Radboud university medical center, Nijmegen, The Netherlands; 3 Department for Health Evidence, Radboud Institute for Health Sciences, Radboud university medical center, Nijmegen, The Netherlands; 4 Centro de Investigaciones y Estudios Avanzados de Maule (CIEAM), Universidad Católica del Maule, Talca, Chile; 5 Centro de Investigación en Neuropsicología y Neurociencias Cognitivas (CINPSI), Universidad Católica del Maule, Talca, Chile; 6 Laboratorio de Neurotoxicología Ambiental, Facultad de Medicina, Universidad Católica del Norte, Larrondo 1281, 1780000 Coquimbo, Chile; 7 Laboratorio de Psiquiatría Translacional, Departamento de Psiquiatría y Salud Mental, Facultad de Medicina, Universidad de Chile, Santiago, Chile; 8 Escuela de Psicología, Facultad de Ciencias Sociales, Universidad Central de Chile, Santiago, Chile; 9 Department of Primary and Community Care, Radboud university medical center, Nijmegen, The Netherlands

**Keywords:** Neurobehavioral performance, Organophosphate, Pesticide, Occupational health, Environmental health, Agricultural work

## Abstract

**Background:**

Previous biomonitoring studies have shown that people in the rural population of Coquimbo, the major agricultural area in northern Chile are being occupationally and environmentally exposed to organophosphate/carbamate (OP/CB) pesticides. Given their harmful effects, this study had two aims; first, to evaluate the effect of cumulative or chronic exposure to OP/CB pesticides on the neurobehavioral performance of agricultural workers and rural inhabitants; second, to determine if changes in the neurobehavioral performance are associated to changes in blood biomarkers of OP/CB pesticides during the spray season, when exposure is higher.

**Methods:**

For the first aim, a cross sectional study of neurobehavioral performance in adult volunteers (men and women, 18–50 years-old, right-handed) was carried out in the pre-spray season. Sampling was done by convenience and a questionnaire was used to categorize participants depending on their level of chronic exposure, as either: occupationally exposed (OE, *n* = 87), environmentally exposed (EE, *n* = 81), or non-exposed controls or reference group (RG, *n* = 100). A neurobehavioral test battery consisting of 21 tests to measure cognitive, motor and emotional state was applied. For the second aim, neurobehavioral measures were taken a second time from EE and OE groups during the spray season, and their exposure corroborated by blood-based biomarker inhibition.

**Results:**

Lower neurobehavioral performance was observed in the pre-spray evaluation of EE and OE groups compared to the non-exposed, OE being the worst performing group. Seasonal exposure impaired performance in both exposure groups on all tests except those on attention and mood. Data modeling of the basal (pre-spray) measurements showed that the level of exposure was the best predictor of performance. During spraying, inhibition of BChE activity in the EE group was the best predictor of low performance in tests measuring logical, auditory and visual memory, inhibitory control of cognitive interference, constructional and planning abilities, executive functions, and motor speed and coordination.

**Conclusion:**

Long-term occupational or environmental exposure to pesticides caused impairment in neurobehavioral functioning, which worsened during the spraying season, mainly in EE. BChE inhibition was the best predictor for seasonal neurobehavioral changes in EE.

## Introduction

In Chile, agriculture contributes to up to 3% of the gross domestic product (GDP) [1]. In 2018, Chilean agriculture was the highest growing sector of the economy reaching an annual rate increase of 5.8%. This is well above that of the global national economy, which expanded by 4%. In addition, exports of US $ 18 billion were made and more than 800 thousand jobs were created (https://www.gob.cl/noticias/ministro-de-agricultura-destaca-el-crecimiento-nacional-del-sector/, accessed 04/21/2020). As well as the number of permanent agricultural workers, every year around 50,000 seasonal workers are recruited for agricultural activities during the harvest season [2].

The Chilean surveillance system of outbreaks of acute pesticide intoxications conducted by the Ministry of Health has reported cases of pesticide poisoning during the spring, which overlaps with the spraying season for insect control in agricultural areas [3]. Pesticide exposure may affect both agricultural workers and individuals from the general population, including children and pregnant women [4–6]. Previous studies conducted in Chilean agricultural communities showed that populations are both occupationally and environmentally exposed to high levels of organophosphates (OP) and carbamates (CB), among other types of pesticides, indicating a need for more effective regulation [6–8].

OP/CB pesticides are mainly used as insecticides. Their mechanism of action is through inhibition of acetylcholinesterase (AChE) in insects, but they also affect the human enzyme [9, 10]. Acute exposure to high concentrations of OP/CB produces an accumulation of acetylcholine (ACh) at central and peripheral synapses triggering cholinergic symptomatology [11, 12].

In order to determine acute exposure, biomonitoring strategies in human populations exposed to OP/CB measure erythrocyte AChE, which is equivalent to the enzyme found in cholinergic synapses [13], and butyrylcholinesterase (BChE), a plasma enzyme synthesized in the liver with similar catalytic properties [14, 15]. Acylpeptide hydrolase (APEH), another enzyme that has been identified as a highly sensitive target for some OPs such as dichlorvos, chlorpyrifosmethyl oxon, and diisopropylfluorophosphate (DFP) [16] has been also studied as a putative biomarker, with some promising results [7, 17].

Contrary to acute exposure, symptoms in populations chronically exposed to low levels of OP/CB are more difficult to identify, partly due to the absence of clear cholinergic symptoms, although subjects can report non-specific symptomatology, including headache, fatigue, insomnia, confusion, and difficulty with concentration [18]. In adults, neurobehavioral impairment due to relatively low concentrations of OPs has been reported particularly in people exposed for more than ten years [19, 20]. In general, there is agreement that chronic occupational OP exposure causes neurobehavioral impairment [21], however this condition often remains undetected in part due to the lack of predictive or diagnostic capacity of cholinesterase biomarkers [22].

In a previous cross-sectional pilot study performed by our group, rural populations exposed to pesticides in Coquimbo, Chile, displayed deficits in executive function, verbal fluency, and visual and auditory memory tests [23]. In this study we report the cumulative and the seasonal effects of pesticide exposure on the neurobehavioral performance of inhabitants of the Coquimbo Region and its association with blood biomarkers.

## Methods

### Study design

**Fig. 1:**
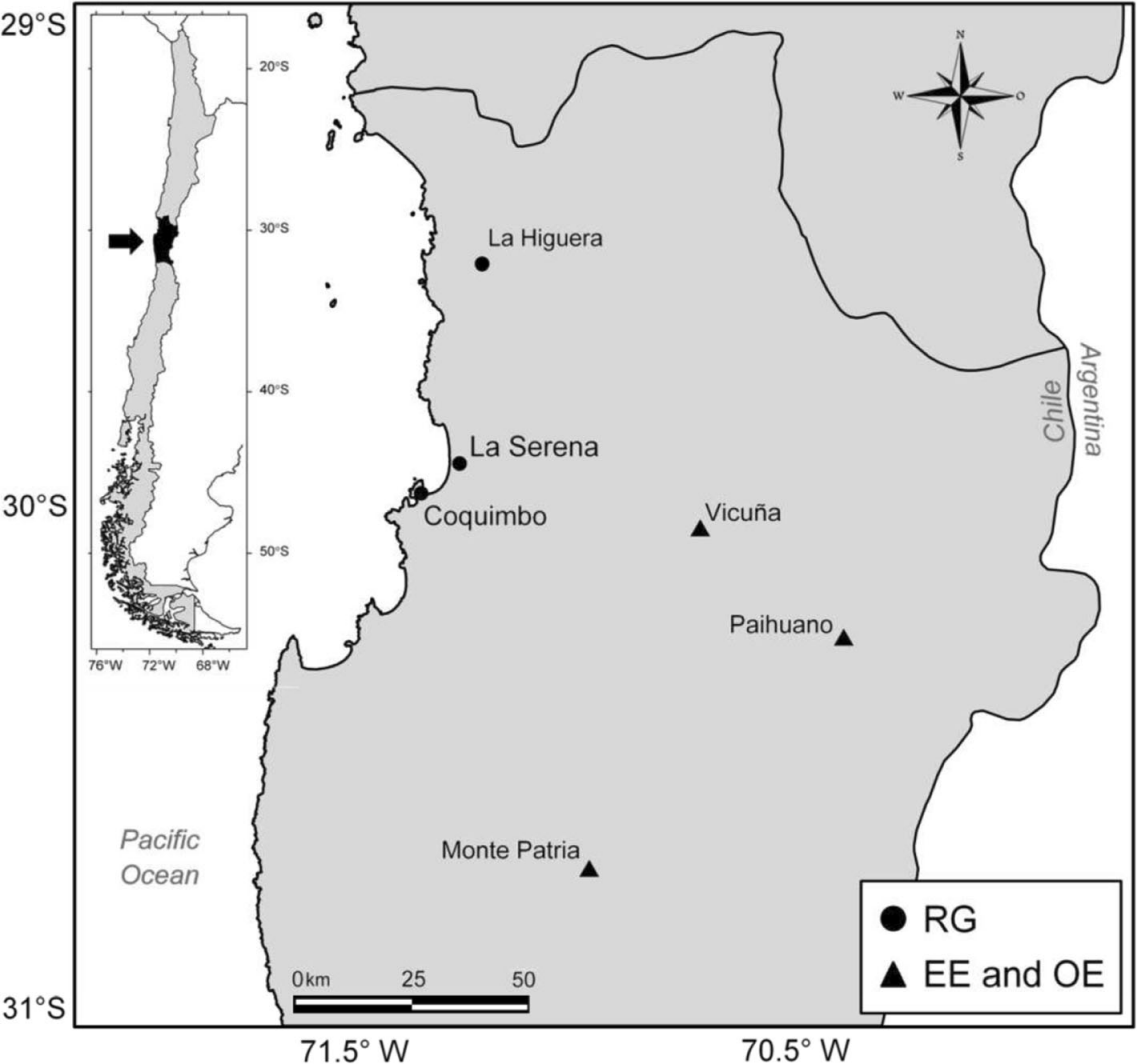
The map shows the geographical locations where the study was conducted. The reference group (RG ●) consisted of workers from urban areas of the cities of Coquimbo and La Serena, and from the rural non-agricultural town of La Higuera. The environmentally- and occupationally-exposed groups (EE and OE ▲) consisted of non-agricultural workers and agricultural workers, respectively; all of them residing in rural areas with intense agricultural activity (Vicuña, Paihuano, Monte Patria). Inner satellite image credit: Google-TerraMetrics, 2018

The study was conducted during 2011–2014 in rural agricultural locations in the Region of Coquimbo, Chile (Fig. 1). The epidemiological design was a serial cross-sectional study in two groups with different forms of pesticide exposure (an environmentally –EE- exposed group and an occupationally -OE- exposed group). A third non-exposed reference group (RG) was used for comparison purposes. The cumulative effect of pesticide exposure on neurobehavioral functioning was estimated in the two exposed groups before spraying and compared with the RG responses. To establish the seasonal effect of pesticide exposure, a second neurobehavioral evaluation plus blood-based biomarkers were measured in EE and OE groups during spraying.

### Sample

As a general requirement, study participants had to be between 18 and 50 years old and right-handed because laterality influences neurocognitive functions. People over 50 years old were excluded due to the cognitive deterioration occurring naturally with age, which could be a confounding factor. A diagnosis of neurological or psychiatric illness and having suffered poisoning events were exclusion criteria for all groups.

Sampling was performed by convenience, and a questionnaire applied to the volunteers that fulfilled the general requirements for classification into the exposure groups. During the interview, sociodemographic and exposure information such as age, years of education, sex and years living in urban, rural or agricultural areas, information regarding morbidity background and subjective symptoms related to cholinergic syndrome was obtained. Additionally, individuals in the OE group were asked about the type of agricultural task performed, the use of personal protective equipment, and their pesticide handling training.

The main inclusion criterion for the OE group was having worked for, at least, the previous 5 years in fruit tree plantations doing agricultural tasks. In contrast to other crops, which have a year-round fumigation schedule, the fumigation of fruit trees (i.e. lemons, oranges or grapes) with OP/CB is carried out only in the spring, allowing clear-cut discriminating between the pre-spray and spray seasons. The environmentally exposed (EE) group were those people who had lived for the last 5 years or more near an agricultural setting (around 1 km or less), had not worked in agricultural activities and had not had direct contact with pesticides. Finally, the RG group consisted of individuals living far away from agricultural settings and with no known exposure to pesticides. Specifically, this latter group consisted of inhabitants of coastal rural areas (fishermen), residents from the rural non-agricultural town of La Higuera and workers (road sweepers and military personnel) from the coastal cities of Coquimbo and La Serena. A diagnosis of neurological or psychiatric illness and having suffered poisoning events were exclusion criteria for all groups. Volunteers that met the inclusion criteria were formally recruited into the study and completed a written informed consent process.

### Field work procedures

The neurobehavioral assessment took up to 3 h to perform. The most difficult or tiring components of testing were presented first, including tests for attention, reaction time, and processing speed, in order to reduce fatigue effects on cognitive performance. Also, a 30 min-break was included at mid-evaluation. To reduce inter-rater bias, the same trained psychologist, supervised by a neuropsychologist, administered the tests to all participants. The same battery was always used for all groups, at both time points. To reduce the possibility of bias due to a learning factor, there was a gap of 3–4 months between evaluations.

All the study procedures were performed according to the Declaration of Helsinki and were approved by the Scientific-Ethics Committee of the Faculty of Medicine, Universidad Católica del Norte, Chile. A complete description of the methodology can be found in the published study protocol [24].

The study lasted three years, with annual recruitment of new volunteers for each study group. EE and OE groups were followed-up over a period of one year in order to cover both the pre-spray and the spray measurements. RG group was measured once at any point in the year.

### Measures

On the day of the evaluation, a blood sample was collected from each study participant in order to measure the activities of classic biomarkers of exposure and/or effect; the enzymes acetylcholinesterase (AChE), butyrylcholinesterase (BChE) and a newly proposed biomarker, the enzyme acylpeptide hydrolase (APEH). The detailed methodology for measurements of biomarker activities is described in Ramírez-Santana et al. [7].

Cumulative pesticide exposure was operationalized as the number of years working in an agricultural setting requiring pesticide use for OE, or number of years living within that agricultural setting for EE.

The selection of the battery of neurobehavioral tests used in this study was based on the core test panel recommended by the World Health Organization (WHO) [25]. Tests that evaluate cognitive function, motor skills, and emotion were included. Cognitive function was evaluated using 16 tests to measure memory (auditory memory and short-term and long-term visuospatial memory), expressive language (nomination), constructional praxis, executive functions (planning, cognitive flexibility, and inhibitory control) and attention. Motor functions were evaluated by determining coordination skills, fine motor skills, and manual dexterity. Finally, emotional status was evaluated to determine the presence of mood disorders, specifically depression and anxiety. A list of neurobehavioral tests is presented in Table 1. The assessment approach was selected based on the criteria of the age of the study participants, acquired literacy skills, and the absence of severe sensory deficits.

**Table 1:** Description of neuropsychological test battery

Function	Neuropsychological test	Aim	Normal cut-off score
General mental status	MMSE	Estimate the severity and progression of cognitive impairment	>24 Rs
Memory	Logical memory I and II (WMS III)	Short and long-term narrative memory assessed with recognition task	≥7 Rs
	Digits span forward	Auditory short-term memory	≥5 Rs
	ROCF memory	Visual memory	≥16 Rs
	1036 A-B and A-B recall	Brief assessment of short-term and long-term visual memory	>20 Rs; >6 recall Rs
Language	WAIS subtest vocabulary	Semantic knowledge and verbal concept formation	≥32 Rs
Attention	WAIS digits span backward	Assess attention span	≥4 Rs
	d2 test	Evaluate the ability of selective and sustained attention	≥Pc 15
	Stroop word-colour and inhibitory control tests	Evaluate the ability of divided attention and resistance to interference	>35 Rs; ≥-10 Rs
	Trail making test A	Evaluate sustained visual attention, sequencing, mental flexibility, visual tracking, and graph motor skills	≥30 ts
	WAIS symbols	Processing speed	>9 Rs
Constructive Praxis	ROCF copy	Visual-constructional ability	>26 Rs
	WAIS subtest block design	Evaluate the ability of visuospatial organization	≥28 Rs
Executive Functions	Tower of London movements and time resolution tests	Assess the ability of executive planning	≥2 Rs; <31 Rs
	WCST perseverative errors	Assess the capacity of executive function, especially mental flexibility	>40 Rs
	Barcelona test categorical evocation animals and words	Assess the accessibility and evocation of lexical and semantic store	≥15 Rs; ≥23 Rs
Psychomotricity	Purdue pegboard test (4 subtest)	Manual dexterity and bimanual coordination	>Pc15
	MOART reaction time (2 subtest)	Go/No Go reaction paradigm	≤180 ms
	MOART finger tapping test (2 subtest)	Motor speed and lateralized coordination	≤50 right hand; ≤45 left hand
Mood Status	BDI-II depression inventory	Measure severity of depression	≤18 Rs
	Hamilton anxiety scale	Determine the presence of symptoms associated with anxiety disorders	≤14 Rs

*Rs* raw score, *ts* t-score, *Pc* percentile, *ms* milliseconds

Number of cigarettes smoked, and average alcohol consumption were measured as covariables. Participants were also asked whether they consumed illicit drugs, the type of drug and frequency of use.

### Statistical analysis

Before statistical comparisons were made, the normality of the data distribution was confirmed using the Kolmogorov–Smirnov test. Continuous variables are presented as mean values and standard deviations (SD). Categorical variables are presented as frequencies and percentages. Data on age, years of education, and alcohol intake (in grams) were compared across exposure groups using analysis of variance (ANOVA). Categorical variables such as sex, smoking habits, and illicit drug use were analyzed using chi-squared (χ^2^) test. To compare the number of years of environmental exposure between both exposed groups (EE and OE), the Mann–Whitney U test was used.

The effect of the cumulative pesticide exposure on the neurobehavioral performance was analyzed using a multivariate quantile regression model for each test. The model was fitted using the R-package *quantreg* and its associated function “rq” [26]. Pesticide exposure was included as a categorical variable (i.e. the exposure groups) and adjusting by gender, age, study years and alcohol consumption, we modeled the median value (tau = 0.5) of each test as a dependent variable of the exposure group, using the RG group as reference. We choose this method since most neurobehavioral variables violate the basic assumptions for conventional linear models, and quantile regression allows for the modeling of data that do not meet conditions for linear regression [26]..

To explore the association between seasonal changes in neurobehavioral performance and biomarker activity, we first established for each biomarker the seasonal change trend of its activity from pre-spray (*t*_*1*_) to spray (*t*_*2*_) season. For this, we calculated the seasonal ratio of each biomarker activity in both, EE and OE individuals, dividing the spray season value by that of the pre-spray (biomarker activity *t*_*2*_/ biomarker activity *t*_*1*_). The ratio value for each biomarker was then analyzed within each exposure group by a one-sample *t*-test. The null hypothesis considered that the expected mean of the population was not statistically different from 1 (no temporal change); in this regard, significant values below 1 suggest biomarker inhibition, while values above 1 would describe the enhancement of the biomarker activity.

A similar procedure was performed to obtain the ratio values between the spray and pre-spray seasons for the neurobehavioral test scores. Both the biomarker and neurobehavioral ratios were used as the input data for quantile regression modeling in order to analyze the extent to which seasonal changes in cognitive performance were explained by changes in biomarker activities. This approach allows for a single value to describe an individual’s performance relative to their baseline, and more importantly, to standardize the different units and magnitudes among variables into a common measure of “relative change”. In this sense the ratio seasonal/baseline > 1 imply increase, < 1 refers to a reduction and 1 means no temporal change in the variable. Depending on the nature of the neuropsychological test, a higher score could mean either better performance, or greater impairment. An increase in the ratio is therefore not necessarily indicative of improvement in the neuropsychological variable. For example, for tests that measure reaction times, ratio increase implies slower processing.

As in the analysis of cumulative effect, all quantile regression models included the sociodemographic covariables identified as potential confounding factors (age, gender, study years and alcohol consumption).

To determine the contribution of each biomarker, we used ANOVA through the function “anova.rq” [26] to compare the amount of variance explained by the full model (i.e. the biomarker activity plus all the covariables) against a “null” model, one without the influence of the biomarker variables. Significant reductions in variance in the full model would imply that the respective biomarker activity is explaining at least part of the model variability.

## Results

A total of 268 participants were enrolled in the study and were distributed as follows: EE, *n* = 81 (at *t*_*1*_) and 78 (*t*_*2*_; three people were lost to follow-up); OE, *n* = 87 (*t*_*1*_) and 78 (*t*_*2*_; nine people were lost to follow-up), and RG, *n* = 100. The sociodemographic characteristics of the study groups are shown in Table 2. The highest proportion of women was observed in the environmentally exposed (EE) group, and the lowest was in the reference group (RG). Individuals in the occupationally exposed (OE) group were older than those in the RG and EE groups. The level of education was higher in the EE group compared with RG and OE groups. No differences were observed in smoking habits, alcohol intake, or occasional drug use. Regarding alcohol intake, > 50% of people in all study groups reported alcohol consumption, with an estimated mean intake of 5 g of alcohol per day. Importantly, both exposed groups (EE and OE) had been living in or near rural areas and agricultural settings for 20.9 ± 14.1 years. Subjects in the OE group had been working for 16.3 ± 8 years in agricultural activities. The proportion of individuals who showed > 30% of inhibition of AChE and BChE activities during the spray season ranged between 15 and 30% (also showed in [7]).

**Table 2:** Socio-demographic characteristics and exposure background of study groups

Variable	RG (*n*=100)	EE (*n*=81)	OE (*n*=87)
Sex (*n*, % of women)^a***^	34 (34.0)	53 (65.4)	45 (51.7)
Age (mean in years ± SD)^b**^	33.8 ± 9.4	34.6 ± 8.0	38.2 ± 8.1
Education (years of study ± SD)^b***^	11.5 ± 2.7	12.5 ± 3.2	9.7 ± 2.5
Smokers (*n*, % yes)^a^	31 (31)	29 (35.8)	32 (36.8)
Grams of alcohol per day (mean ± SD)^b^	5.9 ± 11.6	5.0 ± 13.7	4.1 ±7.8
Drugs (ever tested; *n* and %)^a^	9 (9.0)	3 (3.7)	6 (6.9)
Environmental exposure (years ± SD)^c^	-	20.9 ± 14.1	24.8 ± 14.9
Occupational exposure (years ± SD)	-	-	16.3 ± 8.0
AChE inhibition (%)^d^	-	25.4	23.1
BuChE inhibition (%)^d,#^	-	30.2	15.4

*RG* reference group, *EE* environmentally exposed group, *OE* Occupationally exposed group

^a^Chi-square; ^b^ANOVA *F*_(2,267)_ = 6.7 (age), 21.1 (education); ^c^ Mann - Whitney test; ^d^The values correspond to the proportion of individuals showing

≥ 30% of enzyme inhibition during fumigation

^#^OR^(EE/OE)^ = 1.532 (1.067 – 2.199); *p* = 0.029 (see Ramírez – Santana et al., 2018 [7])

**p < 0.01; ***p < 0.001

**Fig. 2:**
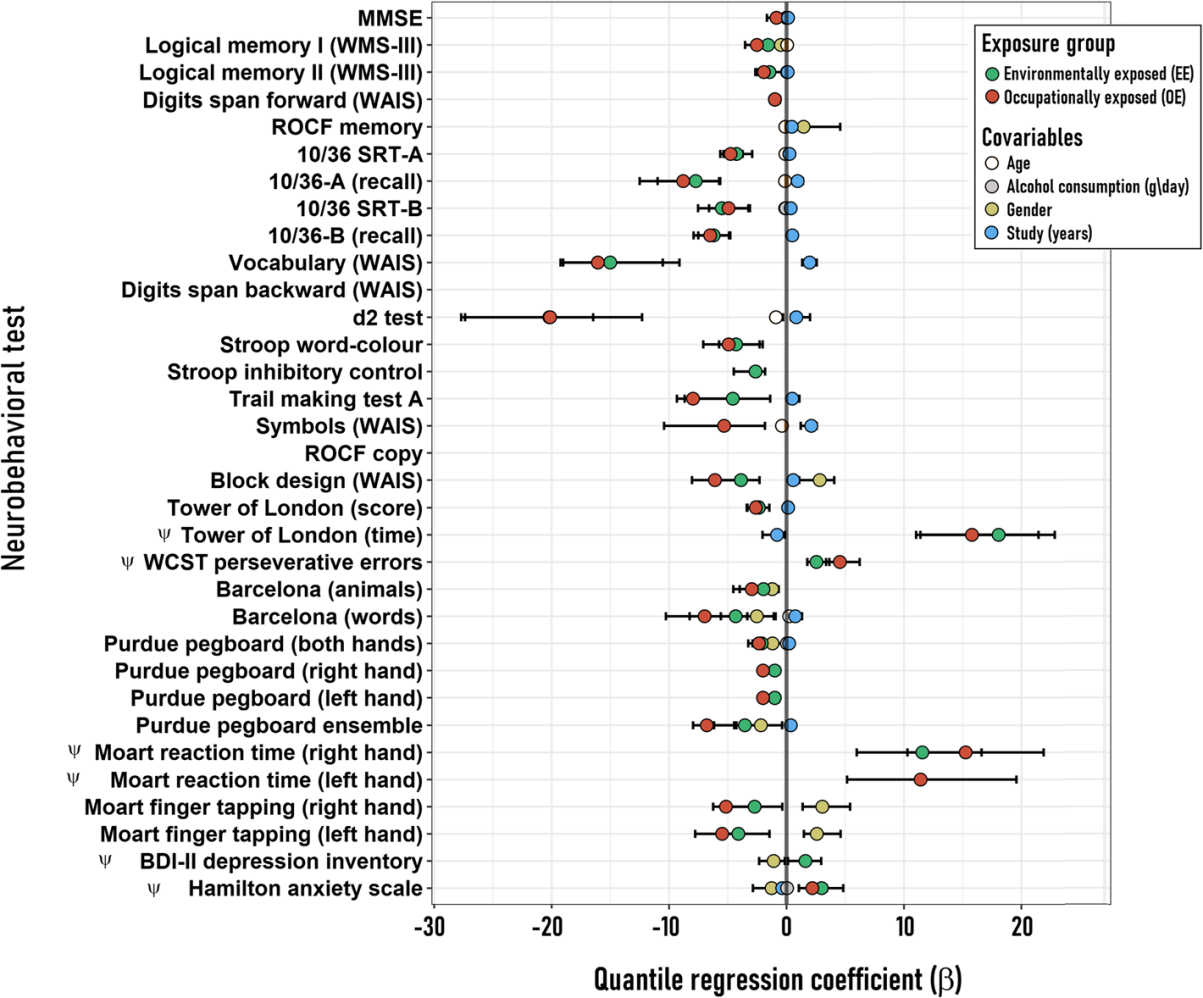
Modeling of the pre-spray neurobehavioral performance. The figure depicts the coefficients (β) and 95% confidence intervals (95% CI) obtained from the multivariate quantile regression analysis performed on each neurobehavioral variable as a function of the exposure group (RG, EE or OE) and a set of sociodemographic covariables (age, alcohol consumption (g/day), study (years) and sex (male – female). For the categorical variables “exposure group” and “sex” the reference in the model was RG and female respectively. Only those coefficients that resulted significant in each model are showed (i.e. their 95 CI’s do not include zero). The scoring of those neurobehavioral variables denoted with a psi symbol (ψ) must be considered inversely-related to the individual performance, this is, higher scores denoted worse performances

Outcomes of the data analysis at the pre-spray evaluation are shown in Fig. 2, depicting the association between long-term pesticide exposure and cognitive performance. While only two tests from the applied battery appear not to be related to the exposure or any covariable (digits span backward and ROCF copy), for the remaining neuropsychological tests, the exposure type (EE or OE) significantly explained the variability in scores (Fig. 2). Moreover, both exposure groups significantly differed from the RG (represented by the mid “zero” line as reference value) in almost all tests. The negative beta coefficients (i.e. less than RG) for those tests in which high scores imply improvement, and positive beta values for tests in which high scores imply deterioration, show that both exposure groups performed worse than the non-exposed across all significant neurobehavioral tests (Fig. 2).

Even considering the effect of confounding factors, data clearly show that both exposure groups are impaired in their neurobehavioral performance since the coefficients and their confidence intervals do not overlap with the reference line. Not surprisingly, the educational level, expressed as years of study, was the most influential covariable in the performance of certain tests. Particularly noticeable is the effect of educational level on WAIS symbols and vocabulary; and on visual memory tests (10/36 A and B). Gender also appears as a factor that influences the performance of some tests. For example, men performed better than women on motor speed tests (MOART finger tapping), but not on fine motor coordination tests (Purdue pegboard test). The visuospatial skills of recall memory and constructional ability (ROCF memory and WAIS block design) were also better represented in men.

Regarding emotional status, men showed less anxiety and depression than women. Alcohol intake contributed to diminished anxiety and age influenced negatively on attention (d2 test), processing speed (WAIS symbols) and some tests of memory. Despite all covariable coefficients depicted in Fig. 2 being significant, they are smaller than those of the exposure groups, and therefore are relatively less important in explaining the variability of the modeled data.

In general, the OE group showed worse cognitive performance than the EE group. The RG displayed the best performance (Fig. S1 A and B). In the supplementary material, detailed results are presented in relation to the neuropsychological performance of each study group at the pre-spray evaluation.

**Fig. 3:**
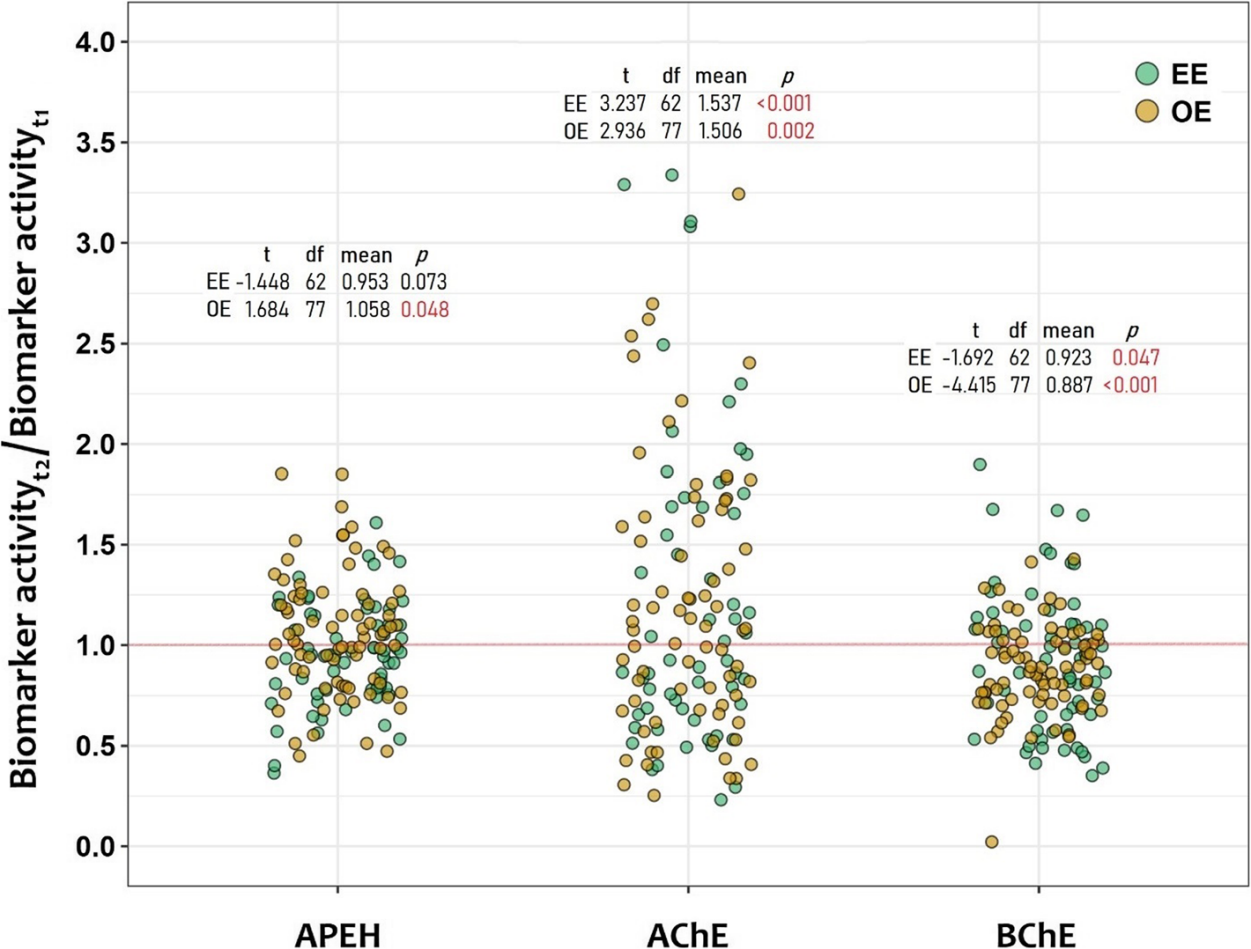
Distribution of the seasonal changes of blood biomarkers (AChE, BChE and APEH) in exposure groups (EE and OE). Each point represents the ratio between biomarker activity during spray and pre-spray for a given individual. Values above 1 suggest increase of the biomarker activity at spray season, while values below 1 implies decrease of the activity. The inner legends show for each group the results for a one-sample t-test (*H*_*o*_: true mean = 1) for the activity value of each biomarker. The red line along 1 represents no temporal change

The inhibitions of AChE and BChE activities were used to demonstrate acute exposure to OP/CB pesticides during the spray season. The three biomarkers behaved differently in relation to their seasonal variation (Fig. 3). For each biomarker, there were individuals showing enhanced or inhibited activity in the EE and OE groups. Nevertheless, only BChE displayed a consistent inhibition in EE as well as in OE, displaying in this last group higher inhibition with a mean ratio value of 0.89 (see methods). By contrast, AChE tended towards enhanced activity during the spray season in both groups. APEH activity differed according to exposure groups, appearing enhanced in the OE group (mean ratio value = 1.06; *p* = 0.048), and not showing significant changes among EE subjects. Detailed information regarding the seasonal analysis of biomarkers is presented in [7].

**Fig. 4:**
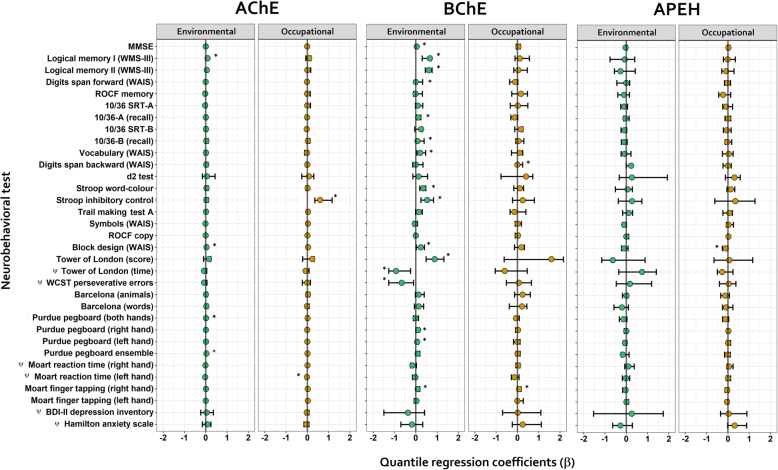
Modeling of the association between seasonal changes in neurobehavioral performance and changes in biomarker activities. Coefficients (β) and 95% confidence intervals (95% CI) were obtained from the multivariate quantile regression analysis performed on each standardized neurobehavioral variable as a function of the change in biomarker’s activities (AChE, BChE and APEH). Green circles represent EE group and golden circles OE group. All models were corrected by the sociodemographic variables (Fig. S3). As the modeled variables were ratios (i.e. they are unitless), significant coefficients (*) can be interpreted as the “percentage of change” in the response variable due to a 1% change in the referred predictor. Coefficient significance was determined by ANOVA analysis between alternative models with and without the respective biomarker activity as explanatory variable (see methods for details). The scoring of those neurobehavioral variables denoted with a psi symbol (ψ) must be considered inversely related to the individual performance, this is, higher scores denoted worse performances

Finally, the analysis of the relation between the seasonal changes on neurobehavioral performance and changes of biomarker activities during the spray season showed that significant associations were primarily observed for changes in BChE activity in the EE group (16 tests out 33). This was true for tests that measure logical, auditory and visual memory, inhibitory control of cognitive interference, constructional and planning abilities, executive functions, and motor speed and coordination (Fig. 4). Significant associations were followed by AChE (4 tests out 33) for logical memory, constructional abilities and fine motor coordination. Poor associations were found for the three biomarkers in the OE group (2 tests with AChE, 2 tests with BChE and 1 test with APEH), highlighting the performance of inhibitory control of cognitive interference, which appeared significantly associated with AChE inhibition. Despite significant, some coefficients were small (< 0.01), while others denoted a stronger relationship between changes in biomarker activity and neurobehavioral performance. This was the case for the Logical Memory tests (I and II), Stroop word-color, Stroop inhibitory control, Tower of London (scoring and time) and the WCST perseverative errors test (Fig. 4). In general, the inhibition of BChE activity during spraying is associated with a worse performance in the EE group. Raw data showing the comparison between the scores before and during spray season for each test is shown in Fig. S2 A and B, and the significant sociodemographic covariables that influence the associations shown in Fig. 4 are depicted separately in Fig. S3.

Sex appeared as the factor that most frequently influenced this relationship. Women were the reference in the multivariate quantile regression analysis, so positive β coefficients indicate that seasonal changes between test score and biomarker activity were higher in men, while negative values imply that greater changes were observed among women. The influence of sex is observed in the association between changes in cholinesterases activities and tests evaluating logical and visual memory, and motor coordination in the OE group; they indicate that men experienced more marked changes than women. In the EE group, women showed larger changes than men in tests of logical and visual memory and attention.

Finally, some tests that measure attention were observed to show an improved performance during spraying. This was the case for the d2 test, which increased from 29.8 ± 24.6 to 34 ± 27.1 (mean ± SD; *p* = 0.011); trail making test A (31.2 ± 7.3 to 32.7 ± 9; *p* = 0.041) and WAIS symbols (8.81 ± 2.4 to 9.27 ± 2.3; *p* = 0.01; Fig. S2A).

## Discussion

The first part of this study demonstrated that to be occupationally or environmentally exposed to pesticides is the main predictor of reduced neurobehavioral functioning. Data modeling was done on standardized test scores with respect to the non-exposed group. In the second part, the seasonal effect of pesticide exposure was explored through modeling of the association between changes in neurobehavioral performance and changes in blood biomarkers during spray season, compared to pre-spray.

The data collected in the first part of the study reflects the cumulative effect of past pesticide exposure reported by participants. Taking into account that they were environmentally or occupationally exposed for more than 10 years and did not report intoxication episodes, they might be considered to be in a long-term low-level exposure status, which is associated with a high prevalence of low-performance in several tests that measure cognitive and psychomotor skills [27]. In the OE group, the results were consistent with the large amount of information available for chronic occupational exposure and reviewed by Ross et al., [28], which concluded that occupational low-level pesticide exposure is associated with impairment in psychomotor speed, executive function, visuospatial ability, working and visual memory.

In a previous pilot study conducted in the same geographical areas, we reported cognitive impairment in a small sample of agricultural workers and in adults residing in areas surrounding agricultural plantations, which we referred to as “indirectly” exposed [23]. The present study supports those results in a larger sample of volunteers, to which a more complete battery of tests was applied, including psychomotor tests and emotional aspects such as anxiety and depression. Together, both studies denote a concerning situation regarding environmental exposure to pesticides, which goes unnoticed, since this type of population is not monitored in epidemiological surveillance programs.

Other studies have addressed the problem of the environmental spread of agricultural pesticides and its impact on the neuropsychological performance in adults of the general population. A study carried out in Greece (Hellenic Longitudinal Investigation of Aging and Diet), has demonstrated that individuals without a diagnosis of dementia who had lived in areas near frequently fumigated fields showed decreased neuropsychological performance compared to those who had never lived in areas near fumigation sites. The most affected domains were language, executive and visuospatial functions and attention [29]. Recently, Paul et al. [30] presented evidence of faster cognitive decline and mortality among older Mexican Americans with a history of chronic environmental OP exposure due to residential proximity to agricultural settings. The association between environmental exposure to pesticides and neurodegenerative diseases has also been demonstrated [31], as well as the relationship with other pathologies such as thyroid dysfunction [32], cancer [33] or reproductive disorders, especially for pesticides with endocrine activity [34]. In summary, there are few cross-sectional, analytical studies considering environmentally-exposed adult populations with cumulative past exposure to OP/CB pesticides, which have associated exposure to the early endpoints of neuropsychological and neurobehavioral impairment. Most of the research that addresses the problem of environmental exposure to pesticides refers to dietary intake or has been carried out in the offspring of exposed pregnant women, children and adolescents, by studying its impact on neurodevelopment [35–38]. On the other hand, as mentioned above, studies in adults have focused on the association between environmental exposure and neurodegenerative diseases [39, 40]. Since cognitive impairment is an initial symptom before possible progression to dementia [41], it is important to detect those who are at risk of developing neurodegenerative diseases in order to apply timely environmental policies with the aim to protect the exposed populations. An interesting question to address in future studies would be how many of the individuals who showed deterioration in some domain or global neurobehavioral impairment will progress to a neurodegenerative disease.

The second part of the study addressed the impact of acute pesticide exposure on neurobehavioral function. Exposure to OP/CB pesticides during the spray season was confirmed by measuring the inhibition of cholinesterases in peripheral blood samples, with respect to the pre-spray value. A significant proportion of study participants in both EE and OE groups displayed inhibition of AChE and BChE activities above the biological tolerance limit (BTL) enforced by Chilean legislation (> 30% of inhibition, Table 2) that establish the threshold for intoxication risk, even though they did not report previous symptoms of OP/CB acute intoxication, which was asked during the interview. In addition to cholinesterases, inhibition of APEH activity was also measured during field biomonitoring. This enzyme is a highly sensitive target for some OPs [17] and has physiological functions in central synapses [42].

In another recently published study, dichotomized data were used for analyzing the association between cholinesterases (AChE and BChE) inhibition above BTL and neurobehavioral impairment by cognitive area (defined by a decrease of 10% in the test performance respect to the pre-spray score). The results indicated that only the EE group showed a large association between BChE inhibition and decreased performance in six of eight cognitive areas evaluated (except for motor coordination and mood). Fewer associations were observed for AChE in the EE and for both biomarkers in the OE [43]. In the present study, biomarker data and standardized tests scores (expressed as ratio values) were modeled in order to find the association between both parameters. As in the previously mentioned study, the modeling yielded similar observations. The majority of significant associations were found in the EE group between BChE inhibition and decreased performance in several tests measuring narrative, auditory and visual memory; visuoconstruction; skills related to executive functions like planning and mental flexibility and fine motor coordination and speed. Less but significant associations were also observed for AChE inhibition and decreased performance in narrative memory, visuoconstruction and fine motor coordination in the EE group.

It is known that BChE activity is a highly sensitive biomarker of exposure to low levels of certain OP/CB pesticides [44] like chlorpyrifos [45] and its oxon metabolite [46]. However, the inhibition of BChE activity is not considered an endpoint for risk assessment as it does not produce the clinical symptomatology of intoxication attributed to AChE inhibition in the cholinergic synapses [47]. Nevertheless, our results indicate that BChE inhibition is a good predictor of low performance on several tests during acute exposure to OP/CB. BChE is expressed in neurons and glia [48], specifically in thalamocortical circuits [49] that have a role in cognition [50]. Thus, it is plausible that its interactions with compounds that modulate its activity have an impact on cognitive performance.

There is general agreement that it is necessary to identify novel biomarkers capable of associating the neurobehavioral effects with the internal dose of OP/CB [22, 51, 52]. Biomarkers related to inflammation, oxidative stress or autoimmunity are plausible candidates [53]. APEH was proposed over fifteen years ago as a putative new biomarker as it is inhibited by some but not all OP pesticides [17], however the data reported here does not demonstrate a significant association between changes in APEH activity and neurobehavioral performance. Interestingly, this enzyme is involved in detoxification pathways of oxidized protein substrates [54, 55] and erythrocyte’s APEH behaves as an endopeptidase towards oxidized peptides [56]. Therefore, future studies could determine the profile of endopeptidase activity rather than the exopeptidase activity measured in this study [7].

Regarding the low association between biomarkers and changes in neurobehavioral performance of the OE group, it is possible to argue that volunteers in this group are already highly deteriorated due to the large number of years that they have been exposed to pesticides, which could mask the effects caused by acute exposures. In fact, our results depicted in Fig. S1 A and B, show a direct dose-response relationship between the degree of exposure reported by group classification and the level of test performance. The graphs show that the worst performance was observed in OE in the majority of tests.

Some authors have reported a high prevalence of anxiety and depression symptoms among individuals with previous pesticide exposure [57–59]. Although both exposed groups showed high scores for anxiety and depression when compared with the RG, these scores decreased significantly during the spray season, indicating an improvement in the emotional domain. This fact demonstrated that the impaired performance observed during spray season was not due to higher anxiety or depression scores.

It is interesting to mention that the performance of tests on attention improved during the spraying season in the EE group. Enhanced scores in processing speed, sustained attention and mental flexibility indicated a tendency towards improvement in attentional performance (Fig. S2A). In agreement with this observation, Fiedler et al. [60] also found some degree of improvement in cognitive performance in children from a rice farm community during the low-use pesticide season, mainly in processing speed. Sustained attentional processes take place in the dorsolateral area of the prefrontal cortex and are modulated by cholinergic neurotransmission [61], however the possibility that inhibition of AChE by OP/CB had an impact in the increase of cholinergic transmission is discarded due to neither AChE nor BChE showing an association with improved performance on tests of attention (d2, trail making test A and WAIS symbols).

As limitations of the study, it is important to highlight that neither the environmental load of pesticides nor the levels of pesticide metabolites in the urine of the exposed populations were measured. In this study, the inhibition of AChE and BChE activities were used as indicators of OP/CB exposure in both agricultural workers and residents of agricultural areas. Undoubtedly, the study groups were exposed to a mixture of different types of pesticides, not all of them cholinesterase inhibitors, therefore the possibility of other targets responsible for the neurophysiological effects observed can not be discarded. Other routes of exposure to chemical residues, such as the dietary intake of vegetables, fruits, or drinking water were assumed to be similar between EE and OE groups.

Regarding the neurobehavioral tests, only six have been validated in Chile, including the mini-mental state examination (MMSE), the digits span forward, the Wechsler Adult Intelligence Scale (WAIS) subtest vocabulary test, the WAIS subtest digit span backward test, the WAIS symbols test, and the WAIS subtest block design test [62, 63]. To address this deficiency, results were compared to non-exposed volunteers living far from agricultural settings as a reference group (RG). Measuring the RG a second time was not considered necessary since cognitive function is expected to be stable, especially within one year, for people younger than 50 years.

## Conclusion

Long-term occupational or environmental exposure to OP/CB were associated with a reduced neurobehavioral functioning in a sample of agricultural workers (OE) and rural inhabitants (EE) of the Coquimbo Region. Seasonal exposure to OP/CB consistenly inhibited BChE activity in the EE and OE groups, and in the EE group this biomarker was the best predictor for reduced performance in logical, auditory and visual memory, inhibitory control of cognitive interference, constructional and planning abilities, executive functions, and motor speed and coordination. The evidence presented here supports the notion that improvements are needed in the regulation and control of the use of pesticides, especially when they are used near residential areas. This should be supported by stricter regulations for the sale and use of pesticides in order to contribute to achieving a higher level of sustainability for health and the environment.

## Supplementary information

**Additional file 1: Figure S1** (panels A and B). Raw scores of neurobehavioral tests measured at pre-spray and their distribution among the exposure groups. Box-plots and bars represent the interquartile distribution of the data in each group. The red line represents the cut-off point determined for each test according to their respective theoretical cut-off scores. For those tests shadowed in grey in panel B, lower scores denote better performances. Asterisks on figures denote the significance of the respective pairwise Wilcoxon Rank-Sum test (* < 0.05; ** < 0.01; *** < 0.001).

**Additional file 2: Figure S2** (panels A and B). Seasonal comparisons of raw scores of neurobehavioral tests within the exposure groups. Blox-plots and bars represent the interquartile distribution of the data in each group. The red line represents the cut-off point determined for each test according to their respective theoretical cut-off score. For those tests shadowed in grey, lower scores denote better performances. Asterisks on figures denote the significance of the respective pairwise Wilcoxon Rank-Sum test (* < 0.05; ** < 0.01; *** < 0.001).

**Additional file 3: Figure S3**. Coefficients (β) and 95% confidence intervals (95% CI) for the sociodemographic covariables. Values of β were obtained from the multivariate quantile regression analysis performed on each neurobehavioral variable as a function of the seasonal change in biomarker’s activities (AChE, BChE and APEH). Green circles represent EE group and golden circles OE group. Only those coefficients that appeared to be significant (i.e. their 95 CI’s do not include zero) in their respective models are showed in the figure.

## Data Availability

The database supporting the results described in the manuscript has been uploaded to ResearchGate as a private file and can be visible upon request (https://www.researchgate.net/publication/335475330_Neuropsychological_data_base).

## Abbreviations

ACh: Acetylcholine
AChE: Acetylcholinesterase
ANOVA: Analysis of variance
APEH: Acyl-peptide hydrolase
BDI-II: Beck Depression Inventory-II
BChE: Butyrylcholinesterase
BTL: Biological tolerance limit
CB: Carbamates
CI: Confidence interval
EE: Environmentally-exposed
GDP: Gross domestic product
HDS: Honestly significant difference
MMSE: Mini-mental state examination
MOART: Multi-Operational Apparatus for Reaction Time
OE: Occupationally-exposed
OP: Organophosphates
OR: Odds ratio
PPE: Personal protective equipment
RG: Reference group
ROCF: Rey–Osterrieth complex figure
SRT: Spatial recall test
WAIS: Wechsler Adult Intelligence Scale
WHO: World Health Organization
