# Supplementary figures and images for: Reduced neurobehavioral functioning in agricultural workers and rural inhabitants exposed to pesticides in northern Chile and its association with blood biomarkers inhibition
Source: Environ Health. 2020 Jul 22;19:84. doi: 10.1186/s12940-020-00634-6 (PMC7374955; doi:10.1186/s12940-020-00634-6)

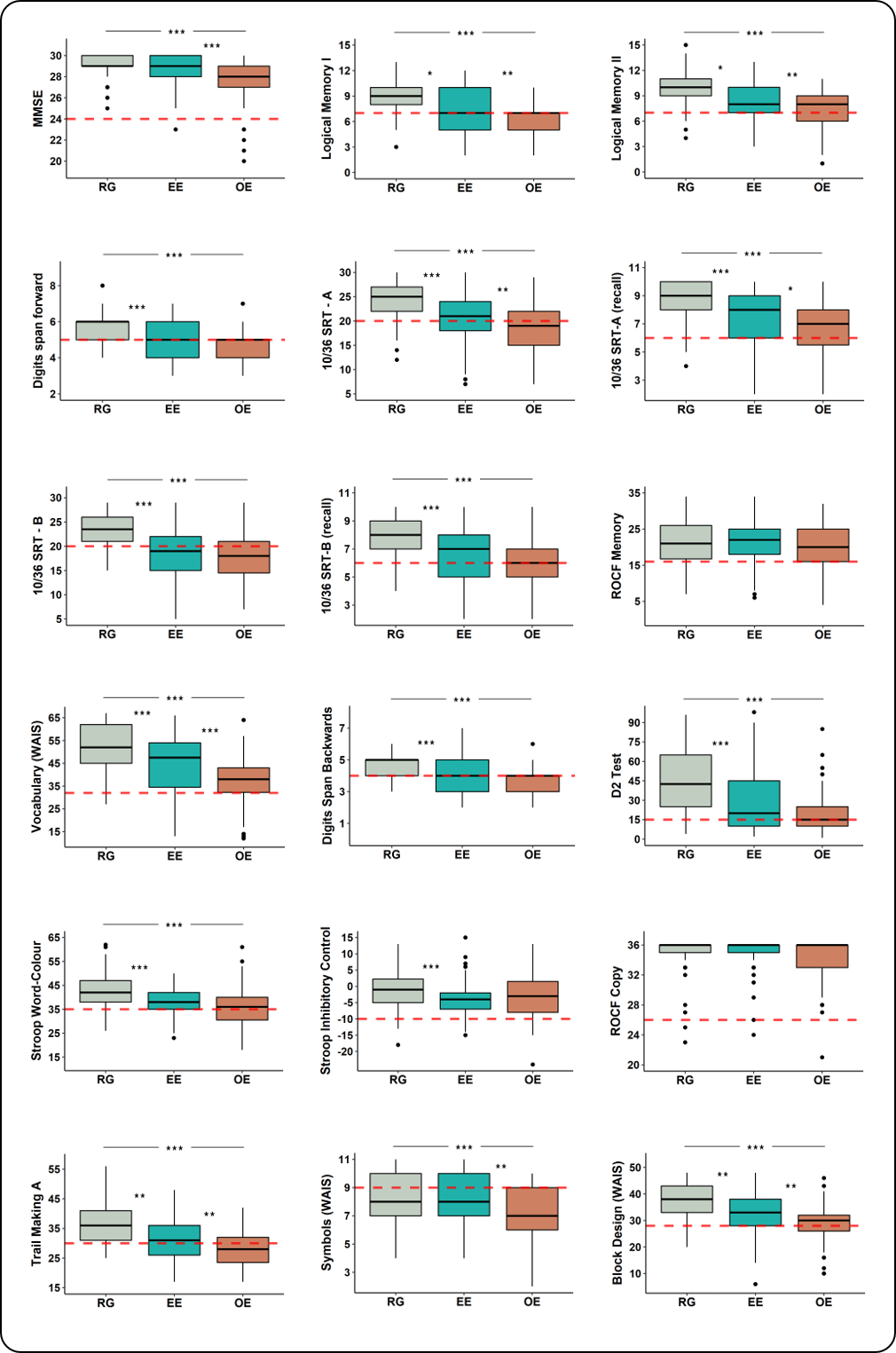

Supplement: Supplementary file 1 — Additional file 1: Figure S1 (panels A and B). Raw scores of neurobehavioral tests measured at pre-spray and their distribution among the exposure groups. Box-plots and bars represent the interquartile distribution of the data in each group. The red line represents the cut-off point determined for each test according to their respective theoretical cut-off scores. For those tests shadowed in grey in panel B, lower scores denote better performances. Asterisks on figures denote the significance of the respective pairwise Wilcoxon Rank-Sum test (* < 0.05; ** < 0.01; *** < 0.001). [file 12940_2020_634_MOESM1_ESM.zip › Fig. S1AR2.png]

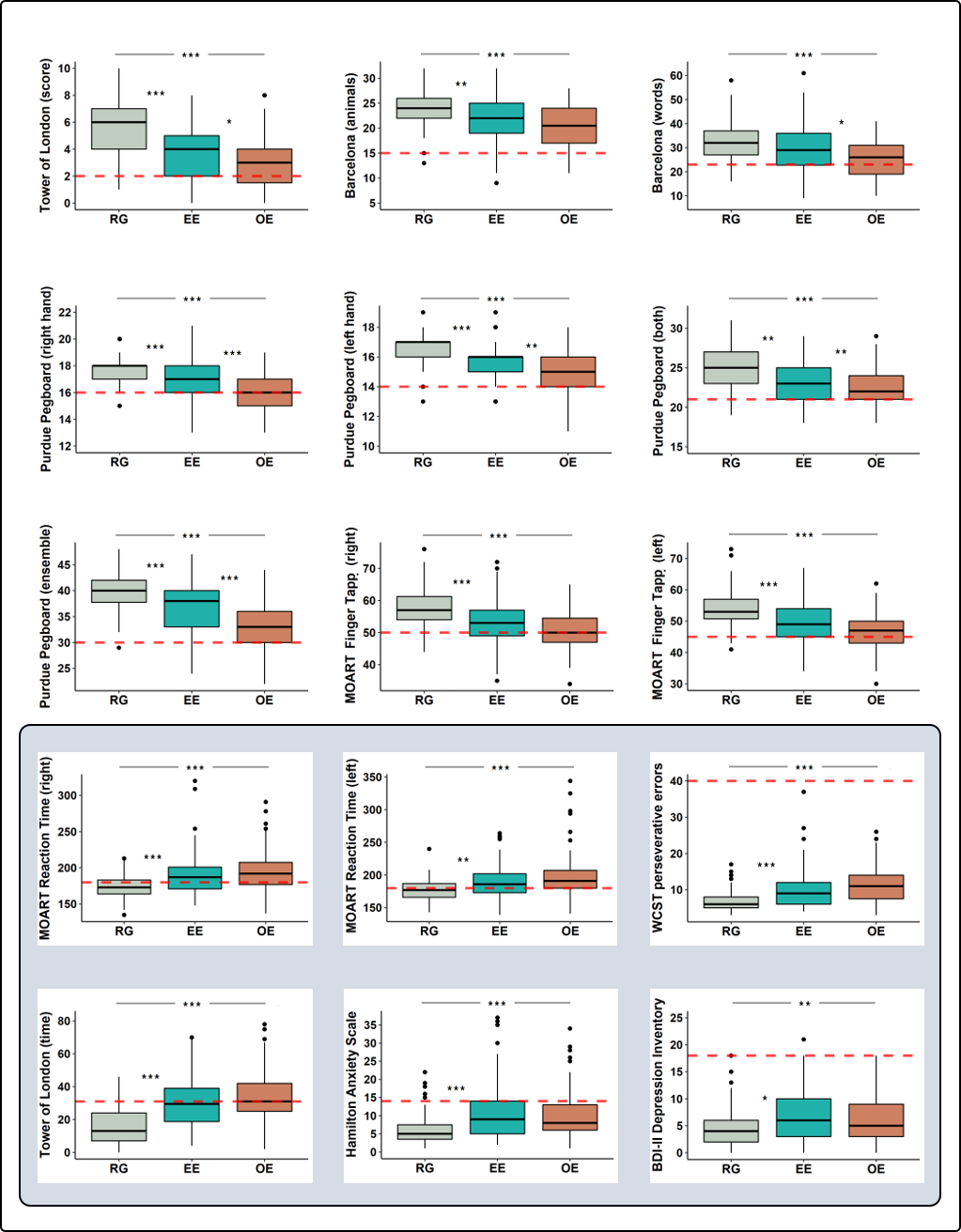

Supplement: Supplementary file 1 — Additional file 1: Figure S1 (panels A and B). Raw scores of neurobehavioral tests measured at pre-spray and their distribution among the exposure groups. Box-plots and bars represent the interquartile distribution of the data in each group. The red line represents the cut-off point determined for each test according to their respective theoretical cut-off scores. For those tests shadowed in grey in panel B, lower scores denote better performances. Asterisks on figures denote the significance of the respective pairwise Wilcoxon Rank-Sum test (* < 0.05; ** < 0.01; *** < 0.001). [file 12940_2020_634_MOESM1_ESM.zip › Fig. S1BR2.png]

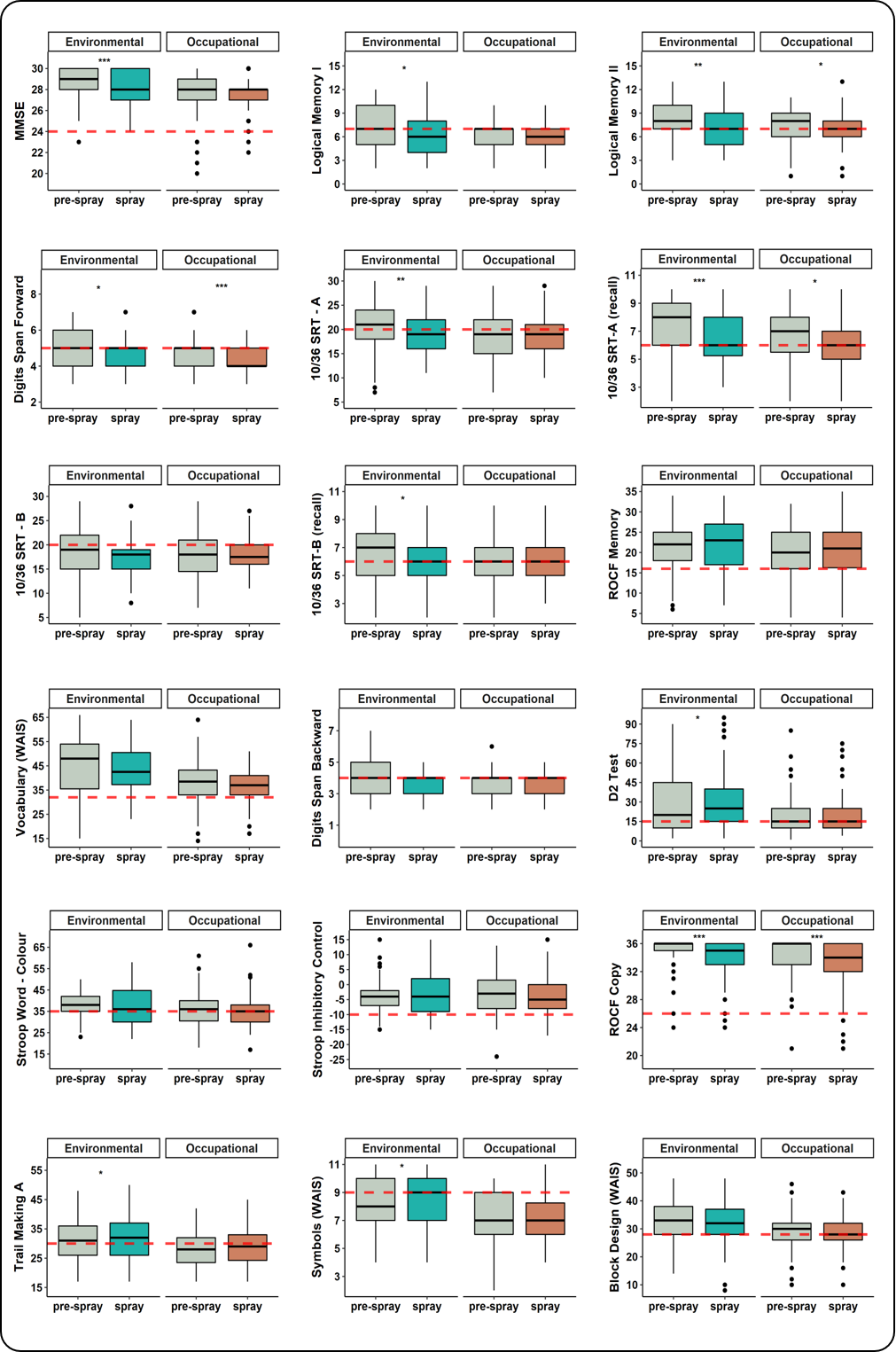

Supplement: Supplementary file 2 — Additional file 2: Figure S2 (panels A and B). Seasonal comparisons of raw scores of neurobehavioral tests within the exposure groups. Blox-plots and bars represent the interquartile distribution of the data in each group. The red line represents the cut-off point determined for each test according to their respective theoretical cut-off score. For those tests shadowed in grey, lower scores denote better performances. Asterisks on figures denote the significance of the respective pairwise Wilcoxon Rank-Sum test (* < 0.05; ** < 0.01; *** < 0.001). [file 12940_2020_634_MOESM2_ESM.zip › Fig S2AR2.png]

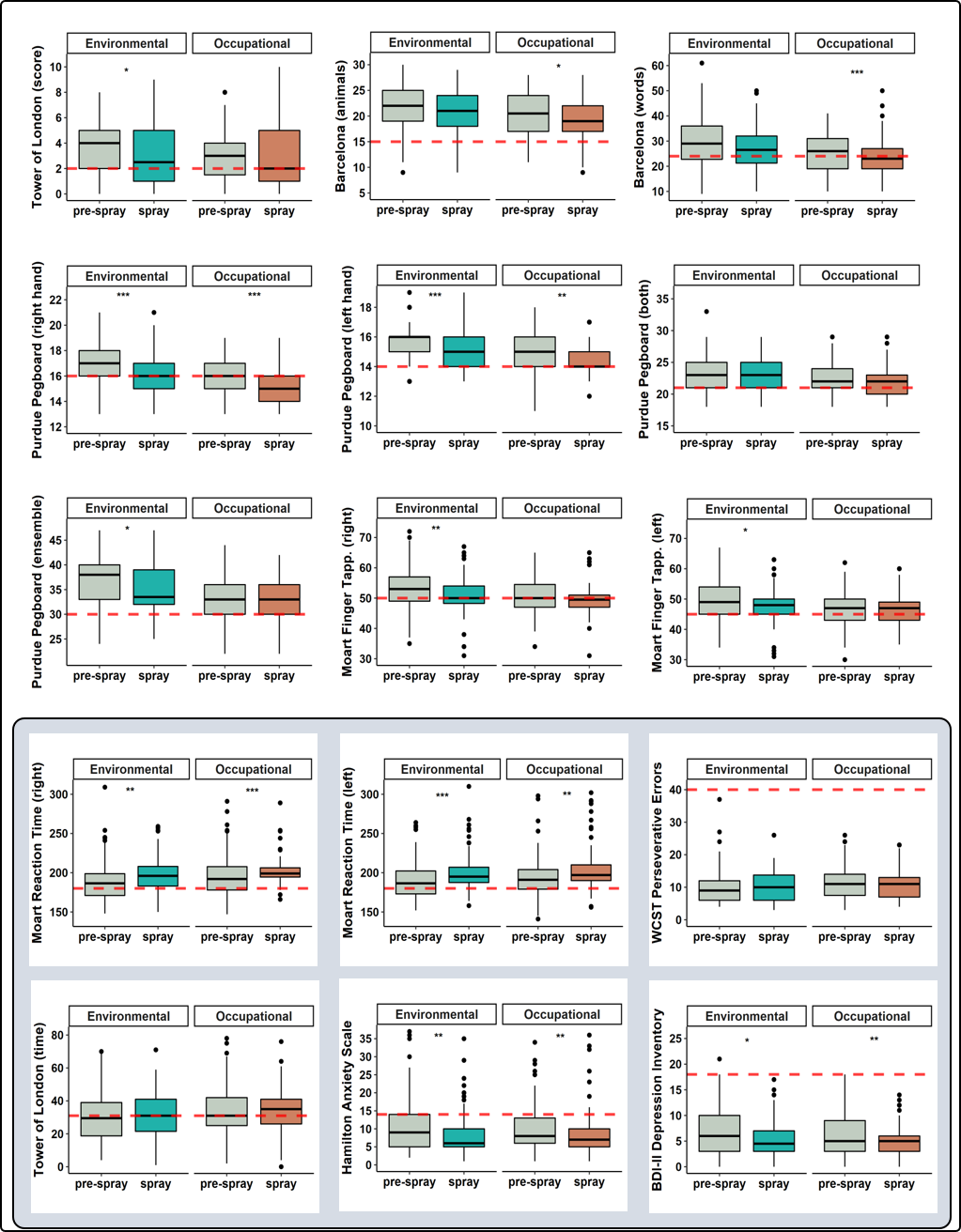

Supplement: Supplementary file 2 — Additional file 2: Figure S2 (panels A and B). Seasonal comparisons of raw scores of neurobehavioral tests within the exposure groups. Blox-plots and bars represent the interquartile distribution of the data in each group. The red line represents the cut-off point determined for each test according to their respective theoretical cut-off score. For those tests shadowed in grey, lower scores denote better performances. Asterisks on figures denote the significance of the respective pairwise Wilcoxon Rank-Sum test (* < 0.05; ** < 0.01; *** < 0.001). [file 12940_2020_634_MOESM2_ESM.zip › Fig S2BR2.png]

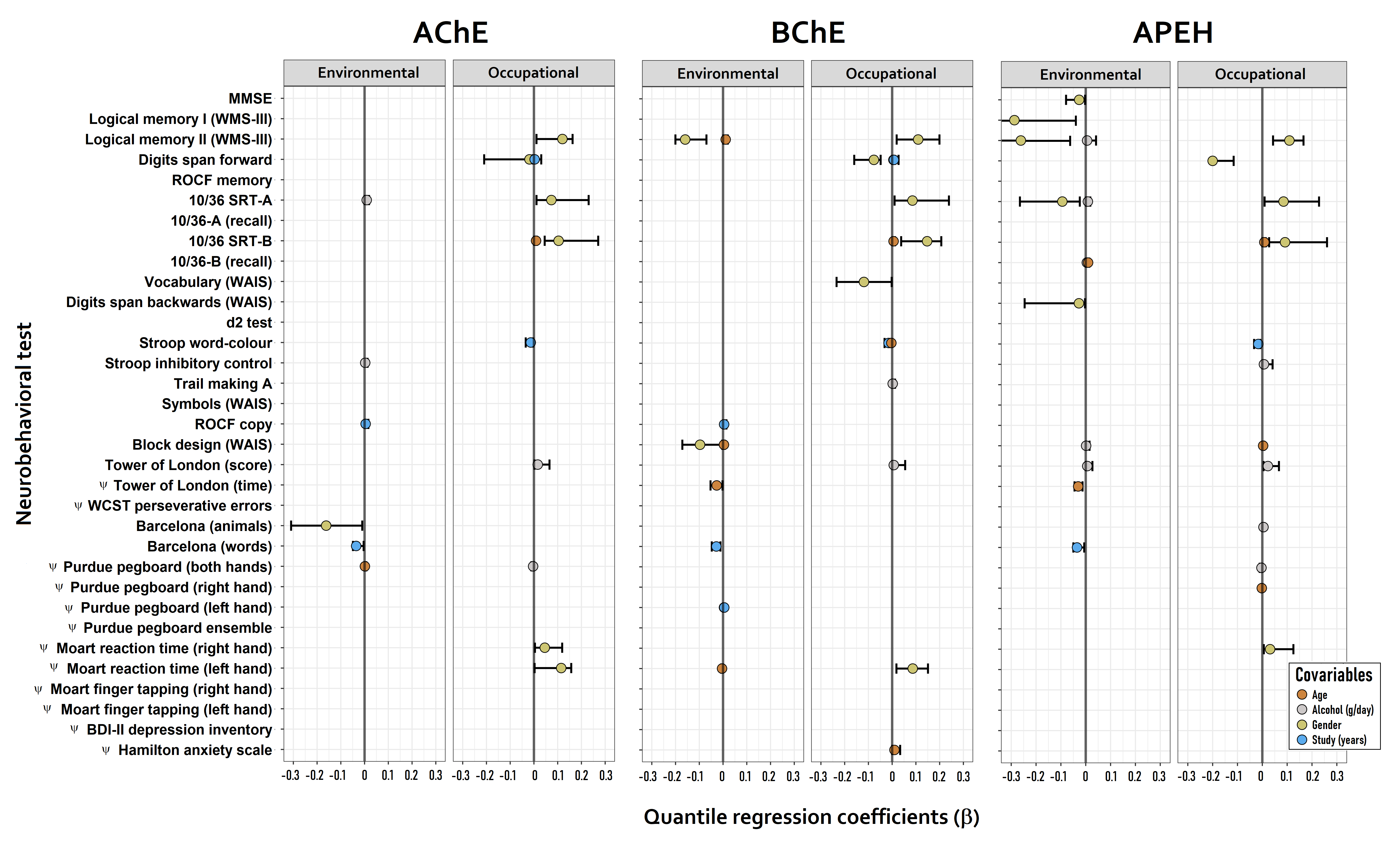

Supplement: Supplementary file 3 — Additional file 3: Figure S3. Coefficients (β) and 95% confidence intervals (95% CI) for the sociodemographic covariables. Values of β were obtained from the multivariate quantile regression analysis performed on each neurobehavioral variable as a function of the seasonal change in biomarker’s activities (AChE, BChE and APEH). Green circles represent EE group and golden circles OE group. Only those coefficients that appeared to be significant (i.e. their 95 CI’s do not include zero) in their respective models are showed in the figure. [file 12940_2020_634_MOESM3_ESM.png]
